# Comorbidities and the referral pathway to access joint replacement surgery: an exploratory qualitative study

**DOI:** 10.1186/s12913-018-3565-0

**Published:** 2018-10-03

**Authors:** Bélène Podmore, Andrew Hutchings, Mary-Alison Durand, John Robson, Sujith Konan, Jan van der Meulen, Rebecca Lynch

**Affiliations:** 1 Department of Health Services Research & Policy, London School of Hygiene & Tropical Medicine, 15-17 Tavistock Place, London, WC1H 9SH UK; 2 Clinical Effectiveness Unit, The Royal College of Surgeons of England, England, UK; 3 Centre for Primary Care and Public Health, Queen Mary University of London, London, UK; 4 University College London Hospitals NHS Foundation Trust, London, UK

**Keywords:** Comorbidities, Access to surgery, Experiences of healthcare professionals, Qualitative design, In-depth interviews

## Abstract

**Background:**

Variation in access to joint replacement surgery has been widely reported but less attention has been given to the impact of comorbidities on the patient journey to joint replacement surgery. There is a lack of consensus amongst healthcare professionals and commissioners about how patients with comorbidities should be referred or selected for joint replacement surgery. It is therefore important to understand the views of healthcare professionals on the management, referral and selection of patients with comorbidities for joint replacement surgery.

**Methods:**

An exploratory qualitative study involving semi-structured interviews with 20 healthcare professionals in England across the referral pathway to joint replacement surgery. They were asked to talk about their experiences of referring and selecting patients with comorbidities for joint replacement surgery. The interviews were audio-recorded and transcribed verbatim. Data analysis followed a thematic analysis approach based on the principles of grounded theory.

**Results:**

In general, the presence of comorbidities was not seen as a barrier to being referred or selected for joint replacement but was seen as a challenge to manage the patients’ journey across the referral pathway. Each professional group, concentrated on different aspects of the patients’ condition which appeared to affect how they managed patients with comorbidities. This implied there was a disagreement about roles and responsibilities in the management of patients with comorbidities. None of the professionals believed it was their responsibility to address comorbidities in preparation for surgery. This disagreement was identified as a reason why some patients seem to ‘get lost’ in the referral system when they were considered to be unprepared for surgery. Patients were then potentially left to manage their own comorbidities before being reconsidered for joint replacement.

**Conclusions:**

At the clinician-level, comorbidities were not perceived as a barrier to accessing joint replacement surgery but at the pathway-level, it may create an implicit barrier such that patients with comorbidities may get ‘lost’ to the system. Further study is needed to explore the roles and responsibilities of professionals across the current orthopaedic referral pathway which may be less suitable for patients with comorbidities.

**Electronic supplementary material:**

The online version of this article (10.1186/s12913-018-3565-0) contains supplementary material, which is available to authorized users.

## Background

Hip and knee joint replacements are two of the most common and effective interventions in the UK [1]. Many patients undergoing joint replacement surgery have at least one comorbid condition (a condition that is present in addition to the joint problem but is an unrelated condition) [2]. As the prevalence of people living with comorbidities increases with age, it is expected that an increasing number of patients with comorbidities will be undergoing hip and knee replacement [3].

In the English National Health Service (NHS), the referral pathway to joint replacement surgery connects primary care, intermediate services and specialist orthopaedic surgeons in secondary care. General practitioners (GPs) are the gatekeepers to secondary care, assessing the patient first in primary care and referring them on to the most appropriate orthopaedic service in secondary care. In certain areas of the country GPs refer patients to intermediate musculoskeletal assessment centres run by physiotherapists or GPs. Introduced in 2006 to reduce waiting times for specialist care, they act as a one-stop-shop for distinguishing patients into those who can benefit from local community services (e.g. physiotherapy, diabetes clinic) and those who need immediate referral to an orthopaedic surgeon [4]. These centres are located in either community or secondary care. In areas without such centres, this triage is undertaken by the surgeons in secondary care.

Evidence suggests that there is wide variation in access to joint replacement surgery in the UK [5–8] which can be partly linked to a lack of consensus about the impact of comorbidities on the risk and benefits of replacement surgery [9, 10]. For example, two studies found that the presence of comorbidities was a reason for some health care professionals to avoid a recommendation for surgery [11, 12]. Studies have also found that different groups of healthcare professionals do not have the same views on who should have joint replacement surgery [5].

In addition, there is also lack of consensus at the commissioning level with some regional commissioners of joint replacement services having sought to limit access to surgery by imposing minimum thresholds for severity of preoperative function [13] and pain [14] and the requirement that a patient’s the body mass index is lower than 30 kg/m^2^ [15]. There is also disagreement about the impact of comorbidities on suitability for joint replacement. However, there is no evidence to support these arbitrary thresholds [16] and the National Institute for Health and Clinical Excellence (NICE), the health authority that develops clinical guidance and quality standards for the NHS in England and Wales, recommends that patient-level factors, including comorbidities, should not preclude patients from being referred to secondary care for joint replacement surgery [17].

Given this lack of consensus, there is a need to get a better understanding of the referral pathway for patients with comorbidities who are candidates for joint replacement surgery and whether their comorbidities has an impact on the care they receive. Coordination between care providers is essential to delivering good quality care for patients with different chronic diseases [18]. Patients with chronic diseases have reported continuity of care as being important when moving across providers and to give them confidence to express their needs to clinicians [19, 20]. Studies have also found that low continuity of care is associated with higher rates of adverse outcomes for patients with multiple chronic diseases [21, 22]. As such it is important to understand the patient journey for patients with comorbidities towards joint replacement surgery.

This exploratory qualitative study investigated the views of GPs, intermediate care professionals, and orthopaedic surgeons on the management, referral and selection of patients with comorbidities for joint replacement surgery.

## Methods

### Sampling strategy

The participants were purposively sampled [23] and included orthopaedic surgeons, GPs and professionals working in intermediate musculoskeletal assessment centres - all professionals who are playing a significant role in either referring or selecting patients for joint replacement surgery in the NHS.

There was an endeavour to include both men and women with a range of years of experience. Orthopaedic surgeons were selected from a list of all orthopaedic surgeons specialising in hip or knee replacements in the NIHR CLAHRC North Thames (North Central and East London, Essex and Hertfordshire) area. Consultant orthopaedic surgeons (senior surgeons who have completed all their specialist training) were contacted via email. GPs were recruited through the local teaching networks using snowballing techniques from a sample of GP practices across the NIHR CLAHRC North Thames area. Intermediate care professionals were recruited, using snowballing techniques, from intermediate services used by GPs and surgeons who had been interviewed.

### Data collection

A semi-structured interview guide was developed and modified slightly for the different roles that the different professionals might have in managing, referring and selecting patients with comorbidities for joint replacement surgery (see Additional file 1). The interview topic guide was developed in consultation with a GP and an orthopaedic surgeon and informed by a scoping literature review on the barriers to accessing joint replacement surgery. The topics included the referral process, how decisions are made to refer or select patient, whether or not and how comorbidities influence clinical decision-making and how these comorbidities affect the patient journey through the orthopaedic referral pathway.

Interviews were conducted by BP and took place face-to-face at the professional’s workplace except for six interviews which were conducted by telephone for the convenience of busy interviewees. Interviews were conducted until no new themes emerged across all professional groups [24]. Interviews were recorded and transcribed verbatim.

### Analysis

Inductive and deductive approaches to data analysis based on the principles of grounded theory were used in order to derive the themes from the responses given by the professionals [25]. The aim was not to generate a theory or a framework. Interview transcripts were initially open-coded to derive broader concepts and to identify major themes based initially on the interview topic guide (deductive coding). Further thematic codes were then drafted inductively and revised regularly through the entire process of analysis through discussion with the members of the research team (BP, RL, MLD, AH and JvdM). This continuous discussion increased coding reliability. The constant comparative method [26] was also used throughout to highlight similarities and differences between healthcare professionals [27]. All data was managed and coded using the qualitative data software program NVIVO. In addition, a first draft of the results section was reviewed by the clinical members of the research team (SK, an orthopaedic surgeon and JR, a GP) and their comments were incorporated.

### Ethical considerations

This study forms part of a larger project investigating the access to and outcomes of hip and knee replacement surgery for patients with different comorbidities. This study received approval from the Health Research Authority NHS Research Ethics Committee (Reference: 16/WA/0241). The participants all signed informed consent and all transcripts and recordings were anonymised.

### Participants

The total sample of this study comprised of 20 English healthcare professionals. This included eight orthopaedic surgeons, seven GPs, and five intermediate care professionals (ICP). Intermediate care professionals were predominantly physiotherapists by training but a small number were also GPs. 13 were men and they had on average been working in their current role for 7 years (range 2–36).

## Results

Regarding the importance of understanding the journey along the referral pathway towards joint replacement surgery for patients with comorbidities, the majority of the professionals reported that the system needed to be improved to better manage patients with comorbidities across the system. As one intermediate care professional explained:

*“…it’s a hole in the NHS provision, if they could get better at stopping patients with long-term conditions crashing and burning, if they could commission something that would help support them so they stayed on a good functional level, all our jobs would be easier” (ICP, Interview 8).*

This quote underlines that while there was an understanding of a need to improve the system for patients with comorbidities the professionals do not know how best to achieve this improvement and whose responsibility it is to make it happen. It also highlights that the professionals perceive managing patients with comorbidities as challenging. This appears to be exemplified in the two major themes that emerged from the data: differences in approaches to managing comorbidities and the professional’s view on whose role and responsibility it is to prepare patients for joint replacement surgery.

### Managing comorbidities

The presence of comorbidities did not preclude the referral or selection of patients for joint replacement. All the professionals indicated that diabetes, chronic obstructive pulmonary disease (COPD) and heart disease were the most common comorbidities which they often found challenging to manage. Each professional group, however, concentrated on different aspects of comorbidity. This different focus influenced how each group of professionals approached the management of patients with comorbidities. GPs focused on the long-term complex care of the patient’s conditions, intermediate care professionals focused on the patients’ eligibility for surgical consultation, and orthopaedic surgeons on the short-term risks of the surgical procedure.

GPs spoke about the complexity of the long-term management of patients’ comorbidities alongside their hip or knee pain. This is perhaps unsurprising as GPs are responsible for the management of the patients before and after surgery. The majority of GPs described their initial assessment of patients presenting with hip or knee pain as being not just about evaluating the risks of the surgery but also about the impact of the comorbidities on the patient’s daily life and the long-term benefits of the hip or knee replacement in terms of improvements in quality of life, pain and disability. One GP explained that he did not think in terms of specific comorbidity categories as one could assume that patients presenting with hip or knee pain were from an age group in which comorbidities were very common (GP, Interview 1). Similarly, several GPs reported general ‘frailty’ in elderly patients as a reason to be concerned about ‘fitness for surgery’ but these GPs seemed to be primarily concerned about the patients’ ability to recover from such an invasive surgery (GP, Interview 3).

GPs reported also having to manage the complexity of ‘multimorbidity’, given the impact different comorbid conditions can have on each other. As one GP described it, core management of joint pain focuses on improving mobility and people with comorbidities struggle even more with mobility. Similarly, the joint pain makes it more difficult for patients to deal with their comorbidities. For some GPs, this complexity did have an impact on the care they believed they could provide in the lead up to joint replacement. For example, as one GP said:

*“If someone’s got severe COPD then offering them physiotherapy may not be an option, the exercise class may not be good enough so there will be comorbidity that will limit what you can and can’t offer in all spectrums, so whether that is medication, exercises, even surgical fitness, you know, all of these come into mind and have to be detailed at the time of consultation.” (GP, Interview 9).*

In contrast, intermediate care professionals, reported focusing the impact that comorbidity has on the patients’ suitability for surgical consultation and the likelihood of patients being selected by surgeons for surgery. Patients, if not sent directly by the GP for orthopaedic consultation, were referred to an intermediate service to be further assessed. The intermediate care professionals reported that, while the presence of comorbidities did not prevent the referral of patients for orthopaedic consultation, a key aspect of their management of patients was the decision about the likely impact that comorbidities have on the risks and benefits of the surgery. As one intermediate care professional explained:

*“I had a patient who had osteoarthritis knee but they’d had a coronary artery bypass graft, they’d had a stroke, they had high blood pressure, they actually hadn’t tried much physiotherapy so we went down the conservative route first, but looking at all of those things and their age, would a surgeon actually want to put them on the operating table for fear that with all of that they might not wake up again. So that’s sort of weighing up the pros and cons of ‘are you going to benefit from this or is there no point in us actually doing the surgery’.” (ICP, Interview 15).*

All intermediate care professionals described risk in terms of the risks of the surgical intervention itself. This may partly reflect the fact that intermediate care professionals reported working more closely with surgeons.

Orthopaedic surgeons focused on the risk comorbidities posed to the surgical procedure itself and the immediate postoperative recovery. Despite this focus on the procedure and postoperative period, they indicated that the presence of comorbidity did not preclude the selection of patients with comorbidities for hip or knee replacement. Orthopaedic surgeons often talked about assessing risks as necessary to avoid the risk of *‘death on the table’* (Surgeon, Interview 6). This risk of death was primarily linked to the impact of comorbidities on anaesthetic risk.

Some surgeons also spoke about what they described as needing to assess the benefits, which they defined as the likelihood of a successful surgery without postoperative complications. Surgeons reported that the likelihood of complications such as cardiac complications was important to consider in the effective management of resources for patients with comorbidities. Patients with multiple comorbidities were labelled as ‘complex patients’ who therefore needed high-dependency beds in case of complications. One surgeon reported that getting a high-dependency bed was challenging. As a result, surgeons explained that in managing patients with comorbidities they had to think not only of the risks of the actual surgical procedure but also the immediate post-operative risks and the logistics of providing care for these ‘complex ‘patients. As one surgeon describes:

*“Last week I did a patient and […] this patient had been put on a list over a year ago and we’d delayed her on three successive occasions, mainly because they needed high-dependency bed...” (Surgeon, Interview 5).*

### Roles and responsibilities

Across the professionals, the theme of roles and responsibilities was central to the discussion about referring and selecting patients for replacement surgery. Differences between professionals groups emerged in how professionals perceived their roles and responsibilities in supporting patients in their preparation for surgery.

GPs indicated that it was not their responsibility to support patients in their preparation for joint replacement by addressing their comorbidities prior to surgery, as they were not clinical orthopaedic experts. They explained therefore that they could not make final decisions on appropriateness for surgery for patients with comorbidities. One GP described the role of GPs more succinctly indicating that it was about *“intervening in modifiable long term risks”* (GP, Interview 11) rather than the short-term risks related to surgery. Another GP reported:

*“I don’t really feel that I’m referring someone for surgery in that kind of way. I kind of more feel like I’m referring them to a specialist who can help them in a special way which might involve surgery. So I don’t, I’m not really in that kind of mindset of this person is going for surgery and I need to be for sure that they’re ready for it” (GP, Interview 3).*

Some GPs mentioned that it was not their role to make a decision about appropriateness for surgery. Others admitted they were not sufficiently informed about what constitutes appropriateness for surgery and therefore let the surgeons make this decision. In addition, some GPs explained that the complexity of the referral system was a barrier to being confidently able to refer and manage patients with comorbidities in preparation for joint replacement surgery.

Intermediate care professionals did not consider it their responsibility to support patients with comorbidities in their preparation for joint replacement surgery. In general, these professionals all suggested that their role was to triage patients referred to them from GPs and not to give an accurate diagnosis or consider fitness for surgery. Again, like GPs, they assigned the role of ‘expert’ to the orthopaedic surgeons. One interviewee explained: *“I’m not a surgeon, I’m not the expert”* (ICP, Interview 15). There was an agreement amongst all intermediate care professionals that their role was very technical and was to ensure that surgeons were sent only those patients who were ‘appropriate’. An appropriate referral was defined as a patient who had tried all non-surgical treatment options and had undergone all investigative tests. All intermediate care professionals suggested that it was important to achieve high ‘conversion rates’, that is, the rate of consultations with the surgeon resulting in a surgical intervention, so that they did not waste a surgeon’s time. Several intermediate care professionals reported that they worked alongside surgeons to improve this conversion rate with the aim of reducing waiting times. They also believed that their role, and the reason intermediate services were introduced, was to relieve the pressure on GPs who were not ‘experts’ either. One intermediate care professional stated:

*“GPs are fantastic, the 13% of their case load is musculoskeletal (MSK) dysfunction and they’re not specialists in MSK, so a lot of the time these patients would be more appropriate to come to us in that we are a cheaper service and our tariff is less but we can give just as good care, but we don’t do the surgery.” (ICP, Interview 16).*

Orthopaedic surgeons defined their role as the ‘expert’ who made the decision about the most appropriate surgical option but were not responsible for supporting patients in their preparation for surgery. One surgeon explained that ideally surgeons would receive only appropriate referrals of patients who needed surgery and were prepared for surgery. At the pre-assessment clinic, surgeons reported that further investigative tests could be ordered if necessary. The majority of surgeons, however, agreed that it was the GP’s main role and responsibility to support patients with comorbidities in their preparation for joint replacement by addressing their comorbidities prior to referral. In order for patients with comorbidities to be prepared for surgery, surgeons explained they needed to be ‘optimised’ – their comorbidities had to be under control. One surgeon reported it was about *“managing those long-term conditions so they don’t delay surgery”* (Surgeon, Interview 5). As he explained, operations were often cancelled due to patients not being ‘optimised’:

*“This week we cancelled a patient on a day surgery, in fact we’d seen her two weeks ago, she had high blood pressure, cancelled her on day surgery, she… hadn’t started on blood pressure medication, sent her back to her GP, “Can you start on medication,”…, a month later she comes back her blood pressure’s even higher than it was the first time around” (Surgeon, Interview 5).*

When patients with comorbidities are assessed by a surgeon and deemed unprepared for surgery the majority of surgeons explained that in most cases they refer patients back to GPs. One surgeon explained there was an incentive to discharge patients as hospitals were penalised if they did not meet the 18-week target from referral to surgery. More than half of the surgeons suggested, however, that GPs may not make re-referrals and patients therefore may be ‘lost to the system’. As a result, these surgeons took it upon themselves to refer patients for further investigations or to other secondary care specialists. They described this as a measure to reduce the waiting time for patients. One surgeon said:

*“I’ll keep them under my review, I won’t discharge them, I’ll bring them back after a few months because I don’t want them getting lost, forgotten about. If I’m not sure, it’s borderline then I might refer to my anaesthetist and ask them their opinion and then they can decide, they may just say yes, that’s fine, just order a few more tests or they may say, yes, I think they need to see a cardiologist for example.” (Surgeon, Interview 20).*

Some GPs and intermediate care professionals reported that patients also had a role in preparing themselves for surgery and this explained why some patients referred back to GPs were not re-referred. They reported that patients were not able to change their lifestyle, to improve their ability to manage their comorbidities, to be prepared for surgery and as a result were never re-referred and never receive the hip or knee replacement. According to one GP:

*“I regard that as basically saying you can’t have the operation because people like her have got to their weight over the course of their life, … most people have very high BMIs so you’re talking about them having to lose some life-changing amount of weight and they don’t do it, so I regard that as just saying no, I’m not going to do your surgery….” (GP, Interview 11).*

## Discussion

The referral pathway towards joint replacement surgery in England generally involves three professional groups: GPs, intermediate care professionals and orthopaedic surgeons. While all professionals reported managing patients with comorbidities across the system as challenging, each group of professionals viewed comorbidities differently and had different opinions about how patients with comorbidities should be managed. This misalignment had an impact on the perceived role and responsibilities of each professional group and how they relate to each other in managing patients who are candidates for joint replacement surgery along the orthopaedic referral pathway. At the individual clinician-level, comorbidities were not perceived as a barrier to surgery but they had an impact on how patients were managed. At the level of the whole referral pathway, comorbidities may create subtle barriers, for example when patients are referred back to GPs and operations may be delayed or never happen.

Each professional group focused on different aspects of comorbidities, and therefore managed patients with comorbidities differently. GPs focused on the long-term impact of comorbidities on the patient’s everyday life and the complexity of multi-morbidity. Intermediate care professionals focused on managing patients with comorbidities through the system and therefore focused on the likelihood of patients being selected by surgeons for surgery. The surgeons focused on the procedure itself and therefore only managed the patients with comorbidity through the surgical intervention but not beyond. Intermediate care professionals and surgeons used similar language when considering comorbidities and describing the management of patients with comorbidities which reflects the close working relationship between the two groups.

Interestingly, while there are differences in focus across all three groups, there is little discussion about comorbidities changing over time [28]. A general assumption appears to be that, once dealt with, the comorbidities will remain constant. This poses a problem in the current system where waiting times for elective joint replacement are long and increasing.

These differences in the way that comorbidities were viewed may point to wider differences between professional groups and how they relate to each other. In addition the different perspectives may lead to fragmented management of patients across the referral pathway to joint replacement surgery indicating the current pathway may be less suitable for patients with comorbidities. This reporting of challenges in coordinating care for patients with comorbidities between primary and secondary care professionals has also been reported in a previous study [29]. Healthcare professionals need to be aware of this potential system failure and more broadly need to consider the impact this has for clinical practice and the care of patients with comorbidities.

GPs and intermediate care professionals did not consider themselves ‘experts’ and therefore did not see it as their responsibility to support patients with comorbidities in their preparation for surgery. Some GPs mentioned this was due to a lack of knowledge which is in accordance with the findings of a survey of GPs about how they make decision about whether or not they should refer a patient for joint replacement surgery. The results of that survey suggest that, on average, they felt only moderately confident about their decisions about who to refer for joint replacement surgery, which was related to their uncertainty about the risks of the surgery [30]. A previous study has found that GPs felt the need for collaboration with ‘specialists’ was even more important for patients with chronic illnesses [31]. This highlights the potential benefits of further guidance on referrals for joint replacement surgery for patients with comorbidities in primary care.

In contrast, surgeons believed that it is the role of GPs to support patients in their preparation for surgery. This tension between GPs and ‘specialists’ in secondary care about their perceived roles and responsibilities has been reported in previous studies [32, 33]. This tension has been reported to be a barrier to delivering coordinated care [29]. Nevertheless, some surgeons, who observed that care was not being provided for patients who were unprepared for surgery, stepped outside the usual clinical pathway and provided the care themselves. These surgeons indicated that they aim to fill a gap in the continuity of care for the patient. In a study investigating the relationship between GPs and hospital consultants across all specialities, hospital consultants were also reported to be filling the gap in continuity of care [34].

Interspersed through the interviews is the observation that patients with comorbidities were getting ‘lost’ as a results of the fragmented management of patients across the referral pathway. Patients with comorbidities who were considered unprepared for surgery by intermediate care professionals or surgeons were reported to be referred back to their GP where often the patients themselves were given responsibility to manage their comorbidities. Many patients however, may not be able to improve the management of their comorbidities [35] and therefore may never receive a joint replacement. It has also been reported in the cancer patient journey that patients are being sent back to their GPs who are not given the information to enable them to provide the continuity of care [36].

A study that carried out a qualitative thematic review exploring the perspectives of patients who were advised to manage their multiple chronic conditions themselves highlighted that access to care when needed is a major challenge [35]. Better support and access to care for patients with comorbidities may therefore play an important role in helping patients to be better prepared for joint replacement surgery.

### Strengths and limitations

The sampling strategy employed for this study allowed us to explore the views of a range of health care professionals across the referral pathway to joint replacement surgery. The aim of this study was not to be representative but to collect extensive varied information in order to obtain an information-rich sample [27]. The study’s limitations in addition to the relative small sample of professionals in each group was its focus on only one region of England which may limit its generalisability to other health systems in other countries. In particular, it is important to note that not every area in England has an intermediate musculoskeletal service within the NHS and therefore the views on the pathway to joint replacement of professionals working in other geographical regions may be different. However, one GP and two surgeons were also recruited from areas where no intermediate musculoskeletal services are operating and their responses to the questions and their understanding of managing comorbidities varied little. We tried to achieve a gender balance but this was not possible as almost all surgeons in our research area were male and almost all intermediate care professionals were female. This might have an impact on the way the participants framed their role. While this study is small in scale, it is unlikely that any of these limitations have affected the results as a diverse set of views were obtained. A larger scale qualitative study, with both patients and healthcare professionals, would be useful however, to further explore the journey along the referral pathway for patients with comorbidities and multimorbidity. Multimorbidity is an area that is underexplored but is an increasing problem for healthcare professionals to manage across the healthcare system [37].

## Conclusions

Research on access to joint replacement surgery has predominantly used quantitative methods to compare characteristics of patients who needed a hip replacement with those who received it [38]. Our qualitative study looked directly at the referral pathway. Patients with comorbidities may access specialist care in terms of surgical consultation but then may not receive a hip or knee replacement. Access to joint replacement surgery seems to be complicated by a fragmented management of patients with comorbidities across the system. This may create an implicit barrier and make the current pathway less suitable for patients with comorbidities.

## Additional file

Additional file 1: Topic guides. The topic guides for the semi-structured interviews with each group of healthcare professionals: Orthopaedic Surgeons, GPs and Intermediate care professionals. (DOCX 23 kb)

## Abbreviations

COPD: Chronic Obstructive Pulmonary Disease
GP: General Practitioner
ICP: Intermediate care professional
MSK: Musculoskeletal
