# Supplementary material for: Comorbidities and the referral pathway to access joint replacement surgery: an exploratory qualitative study
Source: BMC Health Serv Res. 2018 Oct 3;18:754. doi: 10.1186/s12913-018-3565-0 (PMC6171304; doi:10.1186/s12913-018-3565-0)
Supplement: Supplementary file 1 — Topic guides. The topic guides for the semi-structured interviews with each group of healthcare professionals: Orthopaedic Surgeons, GPs and Intermediate care professionals. (DOCX 23 kb) [file 12913_2018_3565_MOESM1_ESM.docx]

# Additional file 1: Topic guides

## 1a. Topic Guide – Orthopaedic Surgeons

**Clarification of details**

- NHS grade
- Number of years practicing/Years of experience
- Type of hospital
- Specialty

**Contextual Factors**

1. Can you tell me who the patients are that are typically referred to you?
   1. E.g. elderly, young with sports injuries, gender, socioeconomic status
   2. How severe are their symptoms?
2. Where are the patients typically referred from?
   1. E.g. musculoskeletal services, GPs, other?
   2. How long have they been waiting to see you?
3. In your opinion what percentage of patients are inappropriately referred and why?
   1. What would you regard as an appropriate referral? What is your conversion rate?

**Assessment/Selection**

1. When a patient is referred to you for hip surgery run me through what you do next.
   1. assessment, diagnosis, selection for surgery
   2. Is it any different for patients with knee patients?
2. What factors do you take into account in your assessment of the patient before selecting them for surgery?
   1. severity of symptoms, duration of symptoms, quality of life
   2. Can you talk through a couple of typical hip and knee patients?
   3. Is it different for hip and knee patients? Do you take into account different factors?

**Long-term Conditions**

1. Do you take into account any Long-term conditions (for example diabetes, Heart disease) that patients may have when selecting patients for surgery? If so, why?
   1. Are LTCS important to take into account?
   2. Hip vs knee patients
2. What the most common LTCs/comorbidities you see?
   1. Can you talk through the last patient you had who had a comorbidity?
3. Are there specific LTCs that you think are especially important to take into account?
   1. controlled vs. uncontrolled LTCs, severity of LTCs

**Other Factors**

1. At what point do you give the go ahead for the patient to undergo surgery?
2. Are there any other factors that influence your decision to go ahead with surgery?
   1. E.g. hospital pressures, financial pressures, other health care professionals, social environment of the patient.
3. How do patients respond when they get selected for surgery?

## 1b. Topic Guide - GPs

**Clarification of details**

- Profession – Partner or salaried
- Number of years practicing/Years of experience
- What type of GP practice (size of GP practice)

**Contextual Factors**

1. How often do you see new patients with hip or knee pain?
   1. E.g. daily, weekly, monthly
   2. prevalence of hip vs. knee pain
2. What kind of patients are they usually? What is a typical patient?
   1. E.g. elderly, young with sports injuries, gender, socioeconomic status
3. Do they come specifically for their hip and knee pain or is it a secondary to another issue?

**Referral Process**

1. When a patient presents with hip or knee pain run me through what you do next.
   1. Assessment
   2. Diagnosis
   3. Referral: where do you send the patients in the first instance?
   4. At what point do you refer patients to be considered for surgery?
   5. Do you do things differently if it is a problem of the Hip vs. the knee?
2. What factors do you take into account in your assessment of the patient before referring them?
   1. Can you talk through a couple of typical hip or knee patients?
   2. severity of symptoms, duration of symptoms, quality of life
   3. Is it different for hip vs knee patients?
   4. In your opinion what are the most important factors?

**Long-term Conditions**

1. Do you take into account any Long-term conditions (for example diabetes, Heart disease) that patients may have in your referral for assessment to surgery? If so, why?
   1. Hip vs knee patients
   2. Are LTCS important to take into account?
2. Are there specific LTCs that you think are especially important to take into account?
   1. controlled vs. uncontrolled LTCs, severity of LTCs

**Other factors**

1. Are there any other factors that influence your decision to refer?
   1. E.g. practice pressures, financial pressures
2. How do patients respond when they get referred for assessment for surgery?

## 1c. Topic Guide – Intermediate care professionals

**Clarification of details**

- Profession
- Number of years practicing/Years of experience

**Contextual Factors**

1. How often do you see new patients with hip or knee pain?
   1. E.g. daily, weekly, monthly
   2. prevalence of hip vs. knee pain
2. What kind of patients are they usually? What is a typical patient?
   1. E.g. elderly, young with sports injuries, gender, socioeconomic status

**Referral Process**

1. When a patient presents with hip or knee pain run me through what you do next.
   1. Assessment
   2. Diagnosis
   3. Referral: where do you send the patients?
   4. At what point do you refer patients to be considered for surgery?
   5. Do you do things differently if it is a problem of the Hip vs. the knee?
2. What factors do you take into account in your assessment of the patient before referring them?
   1. severity of symptoms, duration of symptoms, quality of life
   2. Is it different for hip vs knee patients?
   3. In your opinion what are the most important factors?

**Long-term Conditions**

1. Do you take into account any Long-term conditions (for example diabetes, Heart disease) that patients may have in your referral for assessment to surgery? If so, why?
   1. Hip vs knee patients
   2. Are LTCS important to take into account?
2. What are the most common LTCs/Comorbidities do you see?
   1. Can you talk through the last patient you had who had a comorbidity?
3. Are there specific LTCs that you think are especially important to take into account?
   1. controlled vs. uncontrolled LTCs, severity of LTCs

**Other factors**

1. Are there any other factors that influence your decision to refer?
   1. E.g. service pressures, financial pressures
2. How do patients respond when they get referred to orthopaedic surgeons for assessment for surgery?
